# Design of the BRISC study: a multicentre controlled clinical trial to optimize the communication of breast cancer risks in genetic counselling

**DOI:** 10.1186/1471-2407-8-283

**Published:** 2008-10-03

**Authors:** Caroline F Ockhuysen-Vermey, Lidewij Henneman, Christi J van Asperen, Jan C Oosterwijk, Fred H Menko, Daniëlle RM Timmermans

**Affiliations:** 1 Department of Public and Occupational Health, EMGO Institute, VU University Medical Center, Amsterdam, the Netherlands; 2 Department of Clinical Genetics, Leiden University Medical Center, Leiden, the Netherlands; 3 Department of Clinical Genetics, University Medical Center, Groningen University, Groningen, the Netherlands; 4 Department of Clinical Genetics, VU University Medical Center, Amsterdam, the Netherlands

## Abstract

**Background:**

Understanding risks is considered to be crucial for informed decision-making. Inaccurate risk perception is a common finding in women with a family history of breast cancer attending genetic counseling. As yet, it is unclear how risks should best be communicated in clinical practice. This study protocol describes the design and methods of the BRISC (Breast cancer RISk Communication) study evaluating the effect of different formats of risk communication on the counsellee's risk perception, psychological well-being and decision-making regarding preventive options for breast cancer.

**Methods and design:**

The BRISC study is designed as a pre-post-test controlled group intervention trial with repeated measurements using questionnaires. The intervention-an additional risk consultation-consists of one of 5 conditions that differ in the way counsellee's breast cancer risk is communicated: 1) lifetime risk in numerical format (natural frequencies, i.e. X out of 100), 2) lifetime risk in both numerical format and graphical format (population figures), 3) lifetime risk and age-related risk in numerical format, 4) lifetime risk and age-related risk in both numerical format and graphical format, and 5) lifetime risk in percentages. Condition 6 is the control condition in which no intervention is given (usual care). Participants are unaffected women with a family history of breast cancer attending one of three participating clinical genetic centres in the Netherlands.

**Discussion:**

The BRISC study allows for an evaluation of the effects of different formats of communicating breast cancer risks to counsellees. The results can be used to optimize risk communication in order to improve informed decision-making among women with a family history of breast cancer. They may also be useful for risk communication in other health-related services.

**Trial registration:**

Current Controlled Trials ISRCTN14566836.

## Background

Risk communication aims to improve people's understanding of health risks in order to contribute to informed decision-making. Of particular interest and complexity are risks regarding hereditary or familial cancer, such as breast/ovarian or colon cancer [1]. It is commonly assumed that 5–10% of all breast cancer cases can be attributed to a genetic predisposition, of which the breast cancer genes *BRCA1 *and *BRCA2 *have been identified as most important [2]. Mutations in these genes increase the lifetime risk of breast cancer substantially, e.g. from a 10% population risk [3] to a risk of 60–85% of developing cancer by age 70 [4,5]. However, *BRCA1/2 *mutations account for only a small subgroup of women with a family history of breast cancer. A substantial group of women attending genetic counselling because of their family history have a slightly (11–20%) or moderately (20–30%) increased risk of getting breast cancer. Genetic counselling generally takes place at specialised centres and focuses on the communication of personalised risk information. The aim is to have a well-informed counsellee, who understands both risks and consequences, and acts accordingly [6].

The multiple risks and uncertainty of the risks, e.g. because of the incomplete penetrance of *BRCA1/2 *mutations, introduces complexity into risk communication regarding hereditary and familial breast cancer. Based on these complex risk contexts, decisions have to be made such as whether and when to start mammography screening, and, assuming eligibility, whether to test for mutations in *BRCA1/2*, and subsequent decisions in the case of positive or negative genetic test-outcome. Understanding risks is considered to be crucial for informed decision-making [7], whereas inappropriately high (or low) perceptions of risk can lead to various and potentially harmful decisions, for example overscreening due to overestimation of risk [8]. Overestimation of risk is also shown to be positively correlated with breast cancer worry, which may affect psychological well-being negatively [9]. Previous studies found a high percentage of unaffected women with a family history of breast cancer overestimating their breast cancer risk, although underestimating also occurred (see review [10]). In general, genetic counselling decreases generalized anxiety [11], and significantly improves the accuracy of women's perceptions of their breast cancer risk [11,12], but not necessarily to the correct level [11], or for the long term [12]. Therefore, it is important to identify strategies that will optimize understanding of risk.

Risk communication in genetic counselling is difficult, not only due to the complexity of the risks involved, but also due to problems that counsellors themselves experience in conveying these risks in an understandable way [13]. In addition, the counsellee's understanding and retention of the information given may be problematic. Personal experiences with breast cancer and counsellee's personal characteristics could have mediating effects on the efficacy of risk communication. For instance, the impact of a woman's family history can influence her risk perception prior to counselling through phenomena such as "anchoring" or availability heuristics [14]. Additionally, people with a high need for cognition potentially process risk information more thoroughly and have a higher need for additional risk information compared with people with a low need for cognition [15]. Moreover, women who face difficult decisions with respect to, for instance, genetic testing, may cope differently as a result of their dispositional decision-coping patterns [16].

Regarding the risk communication process, research has shown that the context and format in which genetic risks are presented affect people's risk perception, and their subsequent decisions [17,18]. Although risk presented in numeric terms are precise and preferred by healthy counsellees [19] and patients [20], risks in verbal terms (e.g. "a high risk") are generally more familiar and easier to understand. However, the inherent vagueness of verbal risks leads to substantial variation in their interpretation [21,22]. Risks presented frequentistically (e.g. "1 in 10 cases"), may result in more accurate risk perception and better-founded decisions compared with risks presented in percentages [23]. When frequencies are used, rates (i.e. X in 100) are easier to understand than proportions (i.e. 1 in X) when comparing risks [24]. In sum, both numerical and verbal formats have their pros and cons (see also Lipkus [25]). Several recommendations have been made directed at the use of graphical displays in risk communication [25,26], which may provide helpful support, particularly for persons with low cognitive capacity such as low levels of numeracy [26,27]. Baty et al., for example, reported that graphical displays enabled women to quickly understand their risks of getting breast cancer [28]. However, others have shown that risks presented as icons or pictographs (e.g. population figures in 10 rows of 10) did not result in a better understanding, while having a higher affective impact, and were perceived as larger compared to numerical formats [18,29].

Furthermore, the time horizon in which risks are presented is relevant. Time horizons may raise confusion, especially for long-term cumulative risks such as lifetime breast cancer risks [30]. Short-term risks such as risks for the next ten years may therefore be more meaningful and immediate [30], and may result in less overestimation. Research showed that for the presentation of breast cancer risks, women preferred risk information framed over multiple time horizons (10-year-, 20-year- and lifetime horizons) to risk information framed at one time point [31]. Until now, most studies used hypothetical scenarios in exploring patients' preference for the manner in which risk information is given. However, it is important to discover the best format for communicating cancer risk information in practice tailored to individual counsellees [26].

Several dimensions or outcomes in evaluating the effectiveness of the risk communication process have been distinguished [25,32,33]. Among these dimensions are, firstly, a cognitive evaluation, which refers to risk perception, and understanding and recalling the information provided. Acquisition of knowledge in the context of familial breast cancer comprises of understanding the nature of (familial) breast cancer, knowledge of personal risk factors, understanding the probability of developing breast cancer when having a genetic predisposition, and understanding what actions can be taken to reduce the risk. A second dimension is psychological well-being, including affective reactions and concern, for example, anxiety, (breast cancer) worries, and intrusive thoughts. A third dimension is the decision-making process including a behavioural evaluation, for example with regard to adherence to recommended behaviours (e.g. monthly breast self-examinations, mammography screening) and other decisions such as informing relatives who may be at risk. Finally, another dimension is how counsellees evaluate the information that is provided, which includes the extent to which the risk information is regarded as helpful and clear by counsellees.

Currently, protocols in genetic counselling for breast cancer contain no guidelines for the optimal format of risk communication. Therefore, counsellors present risks in ways that seem right to them despite a lack of evidence to support this [13]. Observational research shows that in breast cancer counselling, risks are given at least half of the time in verbal terms [34,35], and in 25% of the consultations in percentages [36].

In the Netherlands, current information booklets on hereditary breast cancer to support genetic counselling generally present risks, e.g. chance of developing breast cancer, as absolute percentages with a long-term time horizon ('lifetime risk'). In genetic counselling, generally the same formats are used, although one study recently revealed that a time horizon is not often stated in breast cancer counselling [35]. In addition, age-related risks regarding breast cancer counselling are usually not communicated, although in other countries, such as the UK, age-related risks are more commonly used [19].

### Objectives and research questions

The main objective of the present study is to evaluate the effects of different formats of risk communication on the counsellee's understanding and perception of the risk (cognitive outcomes), psychological well-being, and decision-making. In particular, we aim to investigate numerical versus graphical risk formats and lifetime breast cancer risks versus age-related risks. The research questions are:

1) What is the effect of the addition of a graphical format to standard risk information on cognitive outcomes, psychological well-being, and decision-making?

2) What is the effect of the addition of an age-related format to standard risk information on cognitive outcomes, psychological well-being, and decision-making?

3) What is the effect of the combination of both the addition of a graphical format and the addition of an age-related format on outcomes compared to research questions 1 and 2 (i.e. effect modification/interaction)?

4) How do the counsellee's personal experiences and characteristics mediate the effect on the communication of risk?

This article describes and discusses both the design and the content of the intervention of the BRISC (Breast cancer RISk Communication) study, a multicentre controlled clinical trial, to optimise the communication of breast cancer risks in genetic counselling among women with a family history of breast cancer.

## Methods and design

### Study design

The BRISC study is designed as a pre-post-test controlled group intervention trial with repeated measures using questionnaires. The trial is carried out in three of the nine familial cancer clinics in the Netherlands. The Medical Ethics Committees of the VU University Medical Center Amsterdam, the University Medical Center Groningen, and the Leiden University Medical Center approved the protocol in 2005.

### Intervention

**Figure 1:**
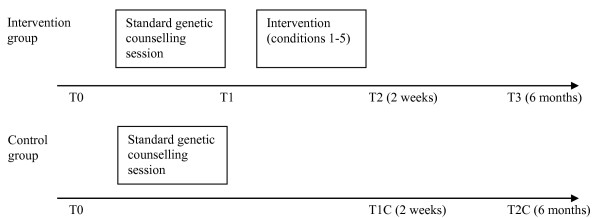
Time planning of the intervention and questionnaires in the intervention group (conditions 1–5) and control group (condition 6).

The intervention consists of one additional consultation by a trained "risk counsellor" immediately after a standard genetic counselling session with a clinical geneticist, a clinical geneticist in training, or non-medical genetic counsellor, henceforth defined as a "genetic counsellor" (Figure 1). Since genetic counselling is seen as a psycho-educational endeavour with both educational and therapeutic elements [37], it is considered unethical to ask the genetic counsellors to present the intervention in a standardized and as objective a way as possible, thereby ignoring the specific needs of the patient. In addition, we assume that besides being unethical, it will not be feasible due to an additional workload for genetic counsellors.

During the additional consultation, risks are communicated in one of five ways (conditions), namely:

1) lifetime breast cancer risk in numerical format (in natural frequencies, i.e. X out of 100),

2) lifetime breast cancer risk in both numerical format and graphical format (i.e. population figures in 10 rows of 10),

3) lifetime breast cancer risk and age-related breast cancer risk in numerical format,

4) lifetime breast cancer risk and age-related breast cancer risk in both numerical format and graphical format, and

5) lifetime breast cancer risk in percentages.

Since the information given during the intervention not only differs from usual care regarding the format of the risks but also to the extent to which the information is structured, two control conditions are included. Condition 5, in which the risk is communicated in percentages as in the standard genetic counselling session (usual care), controls for the effect of giving risk information also in a structured way, enabling us to compare the effects of different risk formats. Condition 6, in which no intervention is given (usual care, henceforth defined as the control group) is used to control for the effect of repetition of the risk information (as compared to condition 5), and will control for Hawthorne effects [38].

**Figure 2:**
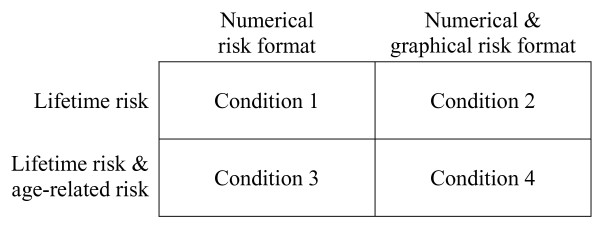
Risk communication formats in conditions 1–4.

**Figure 3:**
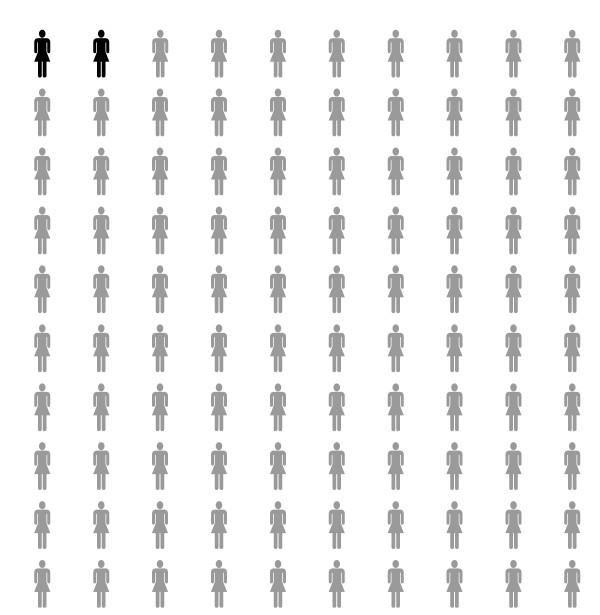
**Example of graphical risk format used in condition 4**. "In the current situation, on average 2 out of 100 women in the Netherlands of the same age as you (40), will develop breast cancer in the next ten years"

As is shown in Table 1, the intervention includes information about risks of developing breast cancer, and having a hereditary predisposition for breast cancer. This includes: risk for an "average" woman in the Netherlands (population risk), risk for women with a *BRCA1/2 *mutation (carriers), and risk for women with the same family history as the counsellee. An example of risk information given in condition 1 is: "On average, 10 out of every 100 women in the Netherlands will develop breast cancer during their lifetime". In conditions 3 and 4, not only the structure of a cumulative risk is explained, but also age-related risks are presented (Figure 2). These age-related risks are computed based on Jonker et al. [39] and comprise breast cancer risks for the next ten years. An example of condition 3 is "In the current situation, on average 2 out of 100 women in the Netherlands of the same age as you (40), will develop breast cancer in the next ten years". In condition 2 and 4 risks are also presented as graphical display (see for example Figure 3).

**Table 1:** Overview of risk information communicated in the intervention

**Population risk:**
Lifetime breast cancer risk: 10 out of 100 for breast cancer patients
Breast cancer risk due to hereditary causes: 5 to 10 out of 100
**Carriers of *BRCA1/BRCA2* mutation:**
Lifetime breast cancer risk: 60 to 80 out of 100
Risk passing a *BRCA1/BRCA2 *mutation to children: 50 out of 100
**Women with the same family history:**
Lifetime breast cancer risk:
- 11–20 out of 100 for women in a low risk category
- 20–30 out of 100 for women in a moderate risk category
- 30–40 out of 100 for women in a high risk category
Risk having a *BRCA1/BRCA2 *mutation: less than 10 out of 100 and specified if more than 10 out of 100

### Usual care

In line with the guidelines of the Dutch Society of Clinical Genetics, genetic counselling generally includes information on the counsellee's lifetime breast cancer risk compared with the lifetime population risk [40]. Based on the individual lifetime risk, three breast cancer risk groups are distinguished: low (11–20%), moderate (20–30%), and high (30–40%). These estimates are mostly calculated using the Claus tables and modified Claus method, and primarily based on a woman's age and family history, including the number of first-degree- or second-degree relatives with breast cancer and the ages of onset of breast cancer [41].

Based on these guidelines, consensus is reached for the study to standardize the content and structure of the standard genetic counselling sessions (usual care) by presenting the woman's breast cancer risk as one of three risk groups, and using percentages for presenting risks. A "counselling checklist" is designed to be used as a guideline during the counselling session. The standard counselling procedures will be discussed with all counsellors involved.

### Study population

Women with a family history of breast cancer who are first-time attendees applying for breast cancer counselling are invited to participate in the study. A family history of breast cancer is defined as having or having had at least one first-degree- and/or paternal second-degree family member with breast cancer. Women are considered ineligible if they are under 18 years of age, have evident psychiatric illness or terminal disease, and are unable to read and write Dutch. Women with a personal history of breast or ovarian cancer are also excluded, because of the complexities in risk counselling of women with a previous history of cancer. Participants are allocated into an intervention or control group (for randomisation procedure, see below). Stratification to first-degree family members is applied to ensure that all family members receive the same condition.

### Sample size and power

The study will be powered on the main outcome measure, namely (change in) risk perception. In order to be able to detect a clinically relevant difference of 10–20% difference between the intervention group and the control group, a sample of 60 women per condition is needed (total n = 360). These calculations are based on a power of 0.80 (1-β) and a significance level of 0.05 (a; two-sided). With an expected drop-out rate of 20%, the sample size for the study is therefore determined at 450. Recruitment and data collection (including follow-up) is anticipated to continue for 31 months.

### Randomisation

Randomisation takes place at 'condition round' level. The inclusion period is divided into condition rounds using a Latin Square design. Every round takes 5 months and switches when a total of n = 75 is reached. The ranking order of conditions (1–5) is varied to correct for learning effect by the risk counsellors. Each condition is given according to the design shown in Table 2. The control group (condition 6) is constructed in addition to each condition round.

**Table 2:** Latin square design of condition rounds over time at the three clinical genetic centres

	**Conditions**^a^				
	months 1–5	months 6–10	months 11–15	months 16–20	months 21–25

Centre A	1	2	3	4	5
Centre B	2	3	4	5	1
Centre C	5	1	2	3	4

^a^. 1) lifetime risk in numerical format (natural frequencies); 2) lifetime risk in both numerical format and graphical format (population figures); 3) lifetime risk and age-related risk in numerical format; 4) lifetime risk and age-related risk in both numerical format and graphical format; 5) lifetime risk in percentages. Condition 6 (control group) is constructed in addition to each condition round.

### Procedure

Women applying for genetic breast cancer counselling are invited to participate by means of an information letter send to their home address. All participants provide written informed consent. In the intervention group, participants receive a self-administered questionnaire at four points in time (T0, T1, T2, T3), the control group receives this at three points in time (T0, T1C, T2C) (see Figure 1):

- T0 (baseline): The women will receive the first questionnaire at home before their first appointment with the genetic counsellor.

- T1 (after standard counselling session): A second questionnaire will be given after the standard genetic counselling session with the genetic counsellor and prior to the intervention (additional counselling with the risk counsellor).

- T2/T1C (2 weeks follow-up): A third questionnaire will be sent to the woman's home address at 2 weeks after the intervention, or a comparable point in time (control group).

- T3/T2C (6 months follow-up): The final questionnaire will be sent to the woman's home address.

A maximum of two reminders is sent to participants not returning their questionnaires. The three risk counsellors (one for each clinical genetics centre) who provide the intervention, have been specifically trained for this study. The intervention lasts for approximately 30 minutes. The risk counsellor is informed about the content of the previous standard counselling session by means of a "checklist after standard counselling", which the genetic counsellor fills in immediately after each session. This checklist includes information about the participant's risks e.g. individual estimated risk of getting breast cancer, also describing the risks formats that were used during counselling. When risks are not yet known, because, for instance, medical records need to be checked, intervention takes place after a second visit. Thus, enforcement of an intervention implies that in the genetic counselling session with the genetic counsellor at least the woman's breast cancer risk group (e.g. low, moderate, high) was communicated.

After the intervention, all participants receive a BRISC brochure in which risks are presented corresponding to the format of the intervention given.

## Measures

### Outcome measures

Outcome measures are classified in four dimensions: 1) cognitive outcomes; 2) psychological well-being; 3) decision-making; 4) evaluation of the intervention (i.e. additional risk consultation). Table 3 shows which variables are measured in which questionnaires.

**Table 3:** Outcome measures of the BRISC study in the different questionnaires

**Variables**	**Instruments**	**Questionnaires**				
		T0	T1	T1C	T2	T3/T2C

**Cognitive outcomes**						
Risk perception						
- numerical scales		X	X	X	X	X
- verbal scales		X	X	X	X	X
- graphical scales				X	X	X
Recall						X
Knowledge of familial breast cancer		X	X	X	X	X
**Psychological well-being**						
Cancer-related worries	CWS	X	X	X	X	X
Cancer-related distress and anxiety	IES			X	X	
Affect	PANAS	X	X	X	X	X
State anxiety	STAI-state	X	X	X	X	X
Anxiety and depression	HADS	X	X	X	X	X
**Decision-making**						
Intention		X	X	X	X	X
Behaviour		X				X
Informed choice	MMIC					
- attitudes towards DNA testing				X	X	
- genetic test-uptake				X	X	X
Decisional conflict	DCS			X	X	X
**Evaluation of the intervention**					X	X
**Personal characteristics**						
Sociodemographic characteristics		X				
Impact of family history				X	X	
Severity of breast cancer	IPQ-RAR			X	X	
Perceived Personal Control	PPC	X		X	X	X
Coping	UCL					X
General anxiety	STAI-trait			X	X	
Need for cognition	NCS			X	X	
Cognitive ability	CRT					X
Decision-making style	Melbourne DMQ					X
Social support	SEC			X	X	

#### Cognitive outcomes

*Risk perception *(primary outcome measure). Table 4 shows the risk perception measures used in the BRISC study. The graphical scales, i.e. Visual Analogue Scales (VAS scales) and graphical display, are only used as a measure after the intervention, to avoid participants of conditions 2 and 4 being triggered before the additional counselling session.

**Table 4:** Risk perception as measured in the BRISC study

**Risk perception**	**Numerical scales**		**Verbal scales**	**Graphical scales**	
	X out of 100	%	Rating scale^a^	Graphical display^b^	VAS scale

**Population risk**					
Lifetime breast cancer risk in the general population	X	X	Very small – very large	X	X
**Risks among carriers**					
Lifetime breast cancer risk in carriers	X	X	Very small – very large	X	X
Risk passing a *BRCA1/BRCA2 *mutation to children	X	X	Very small – very large		
**Participant's risks**					
Perceived risk of having a hereditary predisposition to breast cancer	X	X	Very small – very large Easy to imagine – hard to imagine		
Perceived lifetime breast cancer risk	X	X	Very small – very large		
			Easy to imagine – hard to imagine		
			Very unlikely – very likely		
Perceived age-related breast cancer risk (next ten years)	X	X	Very small – very large Easy to imagine – hard to imagine		
			Very unlikely – very likely		
Appraisal of perceived breast cancer risk, independent of actual risk			Very small – very large Very unfearful – very fearful		
			Not worrisome at all – very worrisome		
Perceived breast cancer risk compared to the average Dutch Woman			Very strongly lowered – very strongly heightened		
**Risks among women with the same family history and age**					
Lifetime breast cancer risk in women with the same family history and age	X			X	X
Age-related breast cancer risk in women with the same family history and age (next ten years)	X			X	X

^a.^Rating scales from (1) – (7) except for: Very strongly lowered (1) – very strongly heightened (9).

^b.^Figure of 100 women.

*Recall *is assessed by asking women to what breast cancer risk group they belong: 1 (not increased risk group) to 5 (very strongly increased risk group). Two additional answering categories are "this has not been discussed" and "don't know (anymore)".

*Knowledge of familial breast cancer *is measured by using a 10-item questionnaire based on questionnaires designed by Lerman et al. [42] and Bluman et al. [43]; adapted and supplemented with items regarding breast cancer (age-related) risk. An expert panel (FM, JO, CA) evaluated the questionnaire using a "true", "false" and "don't know" answering scale, and consensus was reached on the items used (Table 5).

**Table 5:** Knowledge questions of familial breast cancer

	**Items**	True (T)/ False (F)
1	There is more than one gene that can increase the risk of breast cancer	*T*
2	With hereditary breast cancer, breast cancer develops generally more likely at a younger age compared to non-hereditary breast cancer	*T*
3	A father can pass down a genetic predisposition for breast cancer to his daughter	*T*
4	Even if a woman doesn't have a genetic predisposition for breast cancer, her children still can get the genetic predisposition from their grandmother (their mother's mother)	*F*
5	Regular breast cancer screening can prevent the development of breast cancer	*F*
6	A DNA test can determine whether one will or will not certainly develop breast cancer	*F*
7	Genetic inheritance is the main cause of breast cancer in most patients with breast cancer	*F*
8	All women who have a genetic predisposition for breast cancer will get cancer	*F*
9	With ageing, the residual risk of getting breast cancer becomes smaller	*T*
10	The risk of getting breast cancer within the next ten years is equal to the total lifetime risk of getting breast cancer	*F*

#### Psychological well-being

*Breast cancer-related worries *about the risk of developing cancer and the impact of worry on daily functioning is measured by an 8-item adapted Lerman Cancer Worry Scale (CWS) [44].

*Breast cancer-related distress and anxiety *is measured by a Dutch version of the 15-item Impact of Events Scale (IES) [45].

*Affect*, which can be subdivided into positive affect and negative affect (distress), is measured by the 20-item PANAS Scale (Positive and Negative Affect Schedule) [46]. Participants will be instructed to answer the questions in the context of "the past few days".

*State anxiety *is measured by a Dutch version of the 6-item version of the State scale of the Spielberger State-Trait Anxiety Inventory (STAI) [47].

*Anxiety and depression *is measured by a Dutch translation of the 14-item Hospital Anxiety and Depression Scale (HADS), using the timeframe of "last week" [48].

#### Decision-making

*Intention *is measured using 7 self-developed items regarding: breast self-examination; adherence to population mammography screening (above 50 years of age); breast screening by a physician every 6 months; mammography screening every year (own initiative); DNA testing; having a preventive breast operation (mastectomy); informing close relatives. Each item has a 7-point scale: 1 (definitely not) to 7 (definitely), and an additional category "not applicable".

*Behaviour *is measured by 6 items using a timeframe of "the last 6 months", regarding: breast self-examination; adherence to population screening; breast screening by a physician; mammography screening (own initiative); consulting a specialist about a preventive breast operation; preventive breast operation.

*Informed choice with regard to genetic testing*, which is based on knowledge, attitudes, genetic test uptake and value consistency, is assessed using a multi-dimensional measure of informed choice (MMIC) developed by Marteau [49] and extended by Van den Berg [50]. Attitudes towards having a DNA test for breast cancer is measured by two items with a 7-point rating scale ranging from 1 (bad) to 7 (good) and from 1 (useless) to 7 (useful). Genetic test uptake is measured at follow-up (T2/T1C and T3/T2C). To determine value consistency, attitude scores will be combined with test uptake [50].

*Difficulties in decision-making or decisional conflict *are measured by the Dutch version of the Decisional Conflict Scale (DCS) of O'Conner [51].

#### Evaluation of the intervention

The extent to which the additional risk information (intervention) is regarded as useful for decision-making, threatening, and easy to understand was assessed using a 7-point rating scale. Satisfaction with the information provided is measured by a 7-point response format, ranging from 1 (very dissatisfied) to 7 (very satisfied).

#### Personal experiences and characteristics

Several personal characteristics and experiences are measured which may have mediating effects on the efficacy of risk communication.

*Sociodemographic characteristics *included age, level of education, marital status, parent's country of birth, number of children, religious affiliation.

*Impact of family history *is measured using 4 self-developed items regarding: the number of family members that have or have had breast cancer (including the family relationship); perceived impact of this experience by a 7-point scale ranging from 1 (no strong impact) to 7 (very strong impact); the effect of this experience on perceived anxiety regarding getting breast cancer by a 7-point scale ranging from 1 (no strong effect) to 7 (very strong effect); the extent to which the subject "hereditary breast cancer" plays a part in the family by a 7-point scale ranging from 1 (not at all) to 7 (very).

*Severity of breast cancer *is measured by 6 items on the Consequences Subscale of the Dutch version for at-risk individuals of the Revised Illness Perception Questionnaire, that is IPQ-RAR [52].

*Perceived Personal Control *is measured by a Dutch translation of the Perceived Personal Control questionnaire (PPC) [53].

*Coping *is measured by the 19-item Utrecht Coping List (UCL) [54].

*General anxiety *is measured by a 20-item version of the Dutch version of the Trait scale (STAI-form Y) of the Spielberger State-Trait Anxiety Inventory (STAI) [55].

*Need for Cognition *can be described as an "individual disposition to engage in and enjoy thinking", and is measured by an 18-item Dutch version of the Need for Cognition Scale (NCS) [15].

*Cognitive ability *is measured using the 3-item Cognitive Reflection Test (CRT) [56].

*Decision-making style *is measured by the 22-item Melbourne Decision Making Questionnaire (Melbourne DMQ) [57].

*Social support *is measured by the 16-item Social Experiences Checklist (SEC) [58].

### Statistical analyses

Analyses will be performed to estimate the effect of the different interventions (formats) by comparing pre-test- (T0/T1) with post-test- (T2/T1C) outcome measures between the five different intervention groups and the control group, using multivariate analyses. At baseline (T0) and at T1 similar variables will be measured in order to be able to assess the effect of the regular counselling session with the genetic counsellor. Personal variables will be included as covariates. Repeated measures in MANOVA will be used to analyse changes over time and long-term effects (T3/T2C).

## Discussion

Understanding risks is considered to be crucial for informed decision-making. However, inaccurate risk perception is a common finding in women attending genetic counselling regarding breast cancer. Although the literature provides recommendations for the use of frequencies, graphical displays and short-term risks such as age-related risks (i.e. risks for the next ten years) in risk communication, these recommendations are based primarily on research using hypothetical scenarios. In addition, the recommended approaches are not generally used in standard care. The BRISC study is designed to fill the gaps with an evaluation of the effects of different formats of communicating risks to counsellees facing real decisions in a clinical setting. Results will be used to improve risk communication in order to stimulate informed decision-making among women with a family history of breast cancer.

The BRISC study is a field study; in other words, a clinical trial which offers a unique opportunity to evaluate effects of different formats of communicating risks to counsellees facing decisions such as informing relatives who may also be at risk, whether and when to start mammography screening, and, assuming eligibility, whether to test for mutations in *BRCA1/2*.

Quantitative outcome measures are collected using questionnaires at four points in time. The follow-up questionnaire is sent 6 months after the intervention, thereby enabling medium-term effects to be detected. With this comprehensive collected data, it will be possible to clarify the various effects of different formats on four dimensions evaluating the effectiveness of the risk communication process. It is not inconceivable to expect that some formats have positive effects on one of the dimensions and negative effects on another dimension. Additionally, the outcomes of this project will give a better understanding of who, for example, as a function of cognitive capacity, might or might not benefit from different formats.

Two control conditions are introduced in the BRISC study: condition 5 in which the risk information is communicated in percentages as is commonly done in usual care, and a second control condition (condition 6) in which no intervention is given. With these two control conditions, it is possible to distinguish between the effect of repetition and the effect of giving the risks in a structured way on the one hand, and between the effects of giving the risks in a structured way in different formats on the other.

Because the genetic counsellor involved has no role in the inclusion of participants, selection bias is restricted, other than from self-selection. Cluster-randomisation is applied as it is too complex to randomise at the level of counsellees because in that case a risk counsellor is asked to give different risk formats of genetic risks to different counselees on the same day. An exception is made for participants who are close family members.

The risk counsellors who give the intervention are not involved in usual care. This will overcome concerns of contamination bias. All risk counsellors are women, eliminating bias due to gender differences. The Latin Square design and the control group, which is constructed in addition to all condition rounds, both serve to eliminate bias due to time. To achieve uniform risk communication in the genetic counselling sessions, several precautions have been taken (e.g. striving for a standard counselling procedure, "counselling checklist", "checklist after standard counselling"), minimising confounding.

Some limitations of the BRISC study need to be addressed. This study aims at improving women's understanding of the risks, and we acknowledge that there are factors other than risk that can influence the decision-making process. In addition, although several precautions have been taken to achieve an optimal standardisation in counselling and procedures, interactive features must be acknowledged. Another limitation of this study is that it is not possible to blind the genetic counsellors completely because of the nature of the intervention. This applies to the intervention allocation and to the age-related formats due to anticipation of questions by the counsellee. Participants are informed that the BRISC study concerned different kinds of risk counselling and are blinded. Furthermore, although we would have preferred to test an integrated intervention in standard counselling sessions, it is not considered feasible, either ethically or practically, to have the intervention done in a structured way by the genetic counsellors themselves. If one of the formats proves to be most effective, this format could be integrated into the standard counselling session. Since members of the research team provided the additional consultations (interventions), implementation of a format in daily practice will need to receive further attention.

Finally, results may be extended to women with a personal history of cancer, other cancers, and also other diseases and settings, but further study would be required. Imprudent generalization should be avoided, although the BRISC study may provide an indication for risk communication in healthcare in general and cancer genetic counselling in particular.

## Competing interests

The authors declare that they have no competing interests.

## Authors' contributions

CO is responsible for data collection and drafted the manuscript. CO works under direct supervision of DT (Principal Investigator) and LH. DT, LH and FM are co-applicants of the grant. All authors enhanced the design, participated in the standardisation of the counselling sessions, and the development of the intervention. CO, DT and LH designed the questionnaires. CA, JO and FM are involved in recruitment of participants as heads of the family cancer clinics in the clinical genetics centres involved. All authors provided comments on the drafts and have read and approved the final manuscript.

## Pre-publication history

The pre-publication history for this paper can be accessed here:


